# Prioritized High-Confidence Risk Genes for Intellectual Disability Reveal Molecular Convergence During Brain Development

**DOI:** 10.3389/fgene.2018.00349

**Published:** 2018-09-18

**Authors:** Zhenwei Liu, Na Zhang, Yu Zhang, Yaoqiang Du, Tao Zhang, Zhongshan Li, Jinyu Wu, Xiaobing Wang

**Affiliations:** ^1^Institute of Genomic Medicine, Wenzhou Medical University, Wenzhou, China; ^2^Department of Rheumatology, The First Affiliated Hospital of Wenzhou Medical University, Wenzhou, China

**Keywords:** intellectual disability, *de novo* mutations, brain development, gene prioritization, molecular convergence

## Abstract

Dissecting the genetic susceptibility to intellectual disability (ID) based on *de novo* mutations (DNMs) will aid our understanding of the neurobiological and genetic basis of ID. In this study, we identify 63 high-confidence ID genes with *q*-values < 0.1 based on four background DNM rates and coding DNM data sets from multiple sequencing cohorts. Bioinformatic annotations revealed a higher burden of these 63 ID genes in FMRP targets and CHD8 targets, and these genes show evolutionary constraint against functional genetic variation. Moreover, these ID risk genes were preferentially expressed in the cortical regions from the early fetal to late mid-fetal stages. In particular, a genome-wide weighted co-expression network analysis suggested that ID genes tightly converge onto two biological modules (M1 and M2) during human brain development. Functional annotations showed specific enrichment of chromatin modification and transcriptional regulation for M1 and synaptic function for M2, implying the divergent etiology of the two modules. In addition, we curated 12 additional strong ID risk genes whose molecular interconnectivity with known ID genes (*q*-values < 0.3) was greater than random. These findings further highlight the biological convergence of ID risk genes and help improve our understanding of the genetic architecture of ID.

## Introduction

Intellectual disability (ID) is a complex neurodevelopmental disorder characterized by notable deficits in intellectual functioning and adaptive behavior (Ropers, 2010; Musante and Ropers, 2014) with a prevalence of approximately 1% of the world’s population (Maulik et al., 2011). Larger studies have provided compelling evidence that genetic factors are a major contributor to ID and may explain 25–50% of cases, although this association is complicated by extensive clinical and genetic heterogeneity (Vissers et al., 2016). Dissecting the relationship between genetics and ID would advance our understanding of the etiology of this disorder and may offer key information for the development of diagnostics and therapies (Harripaul et al., 2017a).

The whole-exome sequencing (WES) and whole-genome sequencing (WGS) of parent–offspring trios or quartets has established that rare *de novo* mutations (DNMs) play a prominent role in the pathogenesis of severe sporadic ID (de Ligt et al., 2012; Gilissen et al., 2014; Hamdan et al., 2014; Lelieveld et al., 2016). DNMs have been identified as an important source of novel risk genes and provide further insight into the genetic landscape of ID (Hamdan et al., 2014; Lelieveld et al., 2016; Vissers et al., 2016). Screening for recurrent and deleterious DNMs from ever more cohort and family studies has produced a steadily growing number of risk loci and genes associated with ID, such as *DYNC1H1* (de Ligt et al., 2012), *CTNNB1* (de Ligt et al., 2012), *KCNQ3* (Rauch et al., 2012), *DLG4* (Lelieveld et al., 2016), and *PPM1D* (Lelieveld et al., 2016). Statistical analyses of larger cohorts have demonstrated that the candidate genes identified from patients with severe ID often harbor an excess number of loss-of-function (LoF) or functional DNMs with a potentially greater disruptive effect on protein function than expected (Gilissen et al., 2014; Lelieveld et al., 2016). However, due to the extreme genetic heterogeneity of ID, each newly identified gene accounts for only a small proportion of ID cases (Carvill and Mefford, 2015; Vissers et al., 2016). It is therefore still crucial to use available sequencing data to effectively prioritize the causative mutations and candidate genes associated with ID.

Recent functional-network-based analyses, including gene co-expression or physical protein interactions, have shown high functional coherence and connectivity between ID risk genes (Hamdan et al., 2014; Riazuddin et al., 2016; Harripaul et al., 2017b; Shohat et al., 2017). Additionally, Gene Ontology (GO)-based annotations of multiple biological processes in several studies revealed that ID risk genes are significantly associated with nervous system development, RNA metabolism, and transcription, presenting convergent functional features in specific biological pathways (Kochinke et al., 2016). Analyses of the unique spatiotemporal expression patterns of ID risk genes during human brain development indicated that the altered functions of certain specific brain regions were responsible for the range of various clinical ID phenotypes (Parikshak et al., 2015; Harripaul et al., 2017b; Shohat et al., 2017). Therefore, determining ID-associated biological pathways and their expression in the human brain would be of great utility for understanding the pathogenesis of ID (Parikshak et al., 2015; Vissers et al., 2016).

In this study, using TADA statistical model, we identified 63 high-confidence ID genes with *q*-values < 0.1based on all coding DNMs reported to date for ID from currently available trio-based WES/WGS studies. Furthermore, we sought to provide further insight into the pathogenesis of ID by validating these high-confidence ID genes based on a range of function-related analyses. Our analyses showed increased molecular connectivity between strong candidate genes and known ID genes and suggest that these high-confidence ID genes converge on specific brain regions and development stages as well as common biological processes.

## Materials and Methods

### Data Collection and Annotation

All DNM datasets in this study were available from 11 published cohorts for ID and control, and detailed information is shown in **Supplementary Table S1**. In addition, the four background DNM rates (DNMRs), including DNMR-GC (Sanders et al., 2012), DNMR-SC (Samocha et al., 2014), DNMR-MF (Francioli et al., 2015), and DNMR-DM (Jiang et al., 2017), were retrieved from the mirDNMR database (Jiang et al., 2017).

### Annotation of DNMs and Prioritization of ID Risk Genes

By combining the datasets from each study, a total of 1,404 DNMs were collected based on the WES/WGS of 1,027 ID trios and 38,403 from 951 control trios for WGS (**Supplementary Table S1**). We annotated variants using ANNOVAR software (Wang et al., 2010) based on RefSeq hg19 and multiple allele frequency databases (ExAC, UK10K, 1000 Genomes and ESP6500). The functional prediction of missense mutations was performed using 14 integrated tools in ANNOVAR (SIFT, Polyphen2_hdiv, Polyphen2_hvar, LRT, Mutation Taster, Mutation Assessor, FATHMM, RadialSVM, MetaLR, VEST3, CADD, GERP, phyloP100way_vertebrate, SiPhy). After filtering out non-exonic DNMs and common variants with minor allele frequency ≥ 0.001, we focused on 1,392 and 702 *de novo* coding mutations for cases and controls, respectively (**Supplementary Table S2**). We then investigated *de novo* extreme mutations, including LoF [frameshift, indel, stop-gain, stop-loss or splicing single nucleotide variants (SNVs) in coding regions] and missense mutations that were predicted to be damaging by at least eight of the fourteen tools. We then used a Bayesian model of the TADA (TADA-Denovo) to prioritize ID risk genes based on extreme mutations and four background DNMRs, and the TADA *P*-value was adjusted to calculate the *q*-value (He et al., 2013). Genes with a *q*-value < 0.1 for at least three background mutation rates were defined as high-confidence ID risk genes. Known ID genes were derived from three articles (Lelieveld et al., 2016; Vissers et al., 2016; Harripaul et al., 2017b) (**Supplementary Table S3**).

### Conservation and Damage Estimation

We assessed the tolerance of genes to functional genetic variations using the Residual Variation Intolerance Score (RVIS), which measures deviation from the expected amount of common functional variations in genes (Petrovski et al., 2013). Genes with an RVIS score in the top 25% were described as intolerant. The probability of being LoF intolerant (pLI) was derived from ExAC^1^, and genes with a pLI greater than 0.9 were defined as extremely intolerant genes (Lek et al., 2016). Additionally, we defined the ‘hot zone’ as a region that reflects a pLI score greater than 0.9 and an RVIS in the top 25th percentile. The Fragile X Mental Retardation Protein (FMRP) is a polyribosome-associated neuronal RNA-binding protein (Darnell et al., 2011). We collected FMRP targets from two independent data sets, Ascano et al. (2012) (939 genes) and Darnell et al. (2011) (842 genes). CHD8 targets, genes encoding postsynaptic density (PSD) proteins, haploinsufficient genes with predicted haploinsufficient probability greater than 0.9 and constrained genes were derived from previous studies (Huang et al., 2010; Bayes et al., 2011; Samocha et al., 2014; Cotney et al., 2015). We utilized the Fisher’s exact test with correction for multiple comparisons to analyze whether our ID risk genes were enriched in the above gene sets.

### Functional Enrichment Analysis

To characterize the functional convergence of ID, the GO annotations of ID risk genes were determined using DAVID v6.8^2^.

### Network Analysis

The protein–protein interaction (PPI) network used in this study was retrieved from the STRING database^3^ (v10). Analytical data on spatiotemporal enrichment and co-expression were obtained from the HBT database^4^. To construct the co-expression network, we first computed the Pearson correlation coefficient (*r*) between any two genes in the HBT and defined the gene pair as co-expressed if the calculated absolute *r* score was greater than 0.6. We then estimated whether the absolute *r* score of any two gene pairs between 12 novel candidate genes and the 741 known ID genes or 63 known ID genes with *q*-values < 0.3 was greater than 0.6. To prove that the constructed PPI and co-expression networks were not random, we employed a permutation test with 100,000 iterations for genes and their connections. The network was visualized using Cytoscape v3.4.0 (Shannon et al., 2003). Code for permutations performed in **Figure 2** are provided in **Supplementary File S1**.

#### Spatiotemporal Enrichment of ID Risk Genes

In order to gain insight into the spatiotemporal and tissue specific expression of ID risk genes, we used Tissue Specific Expression Analysis (TSEA^5^) (Dougherty et al., 2010) and specific expression analysis across brain regions and development using previously developed tools^6^ (Xu et al., 2014).

#### Weighted Gene Co-expression Network Analysis

As previously described (Langfelder and Horvath, 2008), we performed a weighted gene co-expression network analysis (WGCNA) for ID risk genes using an R package. The expression levels of 60 of the 63 genes across different developmental stages, based on the HBT, were utilized to build gene co-expression modules. The WGCNA clusters the genes using a measure of topological overlap based on the change in the correlation matrix using a power consistent with scale-free topology standards (Zhang and Horvath, 2005). The relevant parameters of the software package were set to 6 for clustering the spatiotemporal expression patterns of a given gene set.

### Results

#### Comprehensive Detection and Prioritization of Candidate ID Risk Genes

**FIGURE 1:**
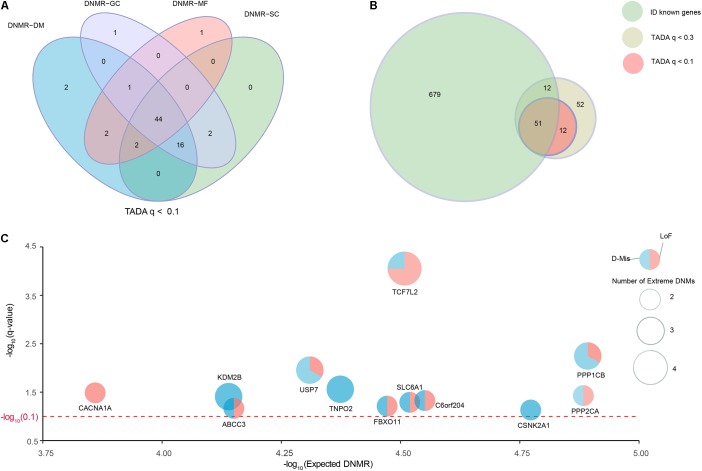
Identification of known and strong candidate ID risk genes. **(A)** Venn diagram denoting the overlap of the number of genes with *q*-values < 0.1 using TADA based on four background DNMRs. **(B)** The intersection of known ID genes, genes with *q*-values < 0.3 and genes with *q*-values < 0.1. Twelve genes with *q*-values < 0.1 were identified as strong candidate ID risk genes. **(C)** Scatter plots depicting the average DNMR for the 12 candidate ID risk genes. The *x*-axis shows the –log_10_ (Expected DNMR) value representing the mutation rate in the TADA program and the *y*-axis represents the –log_10_ (*q*-value) indicating the predicted degree of association (red dotted line, *q*-value = 0.1). The size of each point was weighted with the extreme DNMs number.

We collected a combined cohort of 1,027 ID trios and 951 normal trios through precluding sample redundancies, with a total of 39,807 DNMs from available parent–offspring sequencing studies to comprehensively investigate known and potential ID-associated genes (**Supplementary Table S1**). After excluding non-exonic variants and common variants with MAF ≥ 0.001 based on different public databases (ExAC, UK10K, 1000 Genomes, and ESP6500), we focused on 2,094 DNMs located in the coding regions; these DNMs consisted of 1924 *de novo* SNVs and 170 *de novo* indels (**Supplementary Table S2**). To further optimize and achieve the appropriate power for the discovery of ID-associated genes, we prioritized candidate genes using TADA model based on coding DNMs and four DNMRs (DNMR-GC, DNMR-SC, DNMR-MF, and DNMR-DM). TADA prioritized 71 ID risk genes with *q*-values < 0.1 and 145 with *q*-values < 0.3 using any DNMRs (**Figure 1A** and **Supplementary Figure S1**). Moreover, we found 63 candidate genes with *q* < 0.1 (63/71, 88.7%) that harbored more than one DNM and could be found simultaneously by any three of the background DNMRs, and we defined these as high-confidence risk genes. In addition, 44 candidate genes (44/71, 62.0%) were shared by the four background DNMRs (**Figure 1A** and **Supplementary Table S4**). Of the 145 genes with *q*-values < 0.3 for ID, 127 (127/145, 87.6%) could be found by any three of the background DNMRs, while 92 (92/145, 63.4%) were found by all background DNMRs (**Supplementary Figure S1A** and **Supplementary Table S4**). But no genes showed a *q*-value < 0.1 among the 951 controls (**Supplementary Figure S1B**) and there were only two genes (*SH3D19* and *P2RY14*) with *q*-value < 0.3 (**Supplementary Figure S1C** and **Supplementary Table S4**).

Additionally, we curated 741 well-known ID-associated genes reported in three published studies (**Supplementary Table S3**). After excluding 62 known ID genes of 145 ID risk genes with *q*-value < 0.3, we isolated 12 additional candidate genes with *q*-values < 0.1 and 63 potential candidate genes with *q*-values < 0.3 that harbored DNMs in the ID trios (**Figure 1B**). Of the 12 candidate genes (*q*-values < 0.1), *TCF7L2* had 4 independent DNMs, 4 (*KDM2B, PPP1CB, TNPO2, USP7*) had 3 independent DNMs, 7 (*ABCC3, CACNA1A, CEP85L, CSNK2A1, FBXO11, PPP2CA, SLC6A1*) had 2 independent DNMs (**Figure 1C** and **Supplementary Table S4**). Fourteen generic tools for functional prediction (see section “Materials and Methods”) predicted that approximately 94.7% (18/19) of missense DNMs were damaging (D-Mis). In this study, LoF and D-Mis DNMs were considered extreme mutations. With the exception of one synonymous DNM in *TNPO2*, all DNMs in all other genes were extreme mutations.

#### Functional Co-expression and Physical Interaction Networks of ID Risk Genes

**FIGURE 2:**
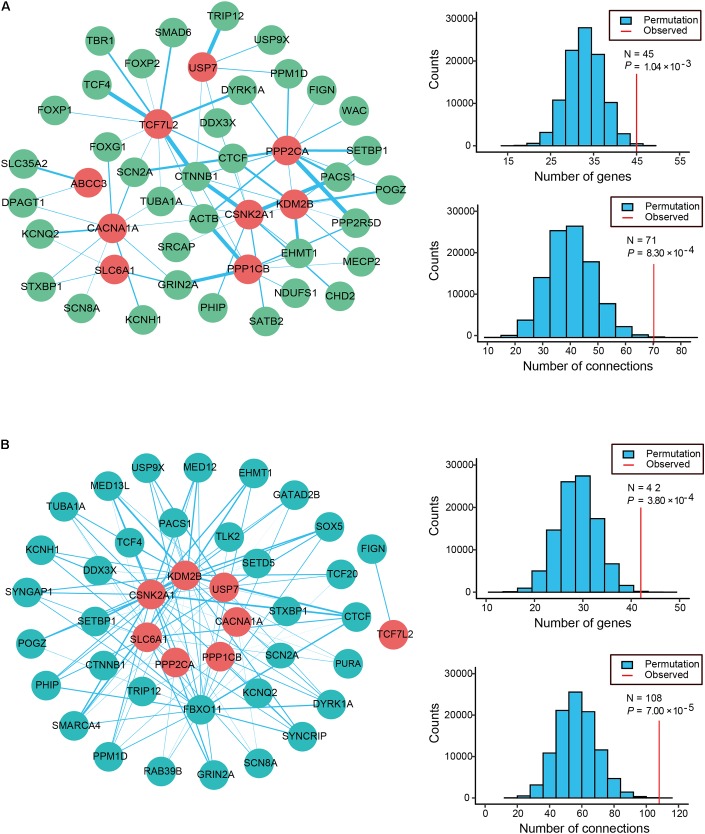
Protein–protein interaction (PPI) and co-expression network analyses of ID risk genes. **(A)** Physical interaction network was created by seeding 12 candidates and known ID genes with *q*-values < 0.3 in STRING. The node color reveals the class of the gene set (known ID genes, dark sea green; candidate ID genes, red), and the thickness of all edges with the color turquoise shows the degree of connectivity (PPI score). **(B)** The co-expression network between the 12 candidate ID genes (cyan) and the 63 known ID genes (firebrick) was analyzed using data from the HBT. Edge (blue line) size indicates the levels of co-expression of the gene pairs estimated by the absolute value of *r* greater than 0.6. The histograms describe the number of genes and connections distributing on the 100,000 interactions. Apart from that, the red vertical lines depict the numbers of observed nodes and connections in the networks. *P*-values are shown in the figures.

Physical interactions often occur between the different causative genes of the same disorder. To evaluate the PPI formed by the 12 candidate genes and the 63 known ID genes with *q*-values < 0.3, we generated an interconnected network using the remarkably comprehensive human protein interactome dataset collected from the STRING database (**Figure 2A**). Our evaluation of the PPI network showed statistical significance for the number of interacting proteins (*P* = 1.04 × 10^-3^) and connections (*P* = 8.30 × 10^-4^) relative to random expectations. Among the interconnected network encoded by 45 genes, 9 candidate genes showed highly likely direct interactions with 36 known ID genes (**Figure 2A**). Strikingly, the 4 genes with the most edges (*PPP2CA, CSNK2A1, TCF7L2, CACNA1A*) interacted with more than 10 known ID genes and *PPP2CA* had the most common gene interactions, associating with 13 known ID genes. Moreover, we found that 11 candidate genes and 337 of the 741 known ID genes formed a significant interaction network which displayed more connections than random expectation (*P* = 1.00 × 10^-5^ for genes; *P* = 1.00 × 10^-5^ for connections; **Supplementary Figure S2A**).

To further explore the functional relevance of the 12 candidate genes and the known ID genes, we performed a co-expression network analysis based on the spatiotemporal transcriptome data set of the developing brain found in the Human Brain Transcriptome (HBT) database. We observed the clear co-expression of novel candidate genes and the known ID genes, as demonstrated by their absolute *r*-values greater than 0.6 (**Figure 2B**). Eight of these candidate genes were more frequently co-expressed with 34 of the known ID genes than would be expected by chance (*P* = 3.80 × 10^-4^ for genes; *P* = 7.00 × 10^-5^ for connections; **Figure 2B**). A further analysis of the network revealed that the four genes with the most edges (*KDM2B, CSNK2A1, FBXO11*, and *SLC6A1*) interacted with more than 15 known ID genes. Furthermore, 11 candidate genes were more frequently co-expressed with 292 known ID genes than those observed in randomly permuted networks (*P* = 3.00 × 10^-5^ for genes; *P* = 9.80 × 10^-4^ for connections; **Supplementary Figure S2B**). Our PPI and co-expression data provided support for the biological relationship between the 12 candidate ID genes.

### Functional Characteristics and Evaluation of ID Risk Genes

**FIGURE 3:**
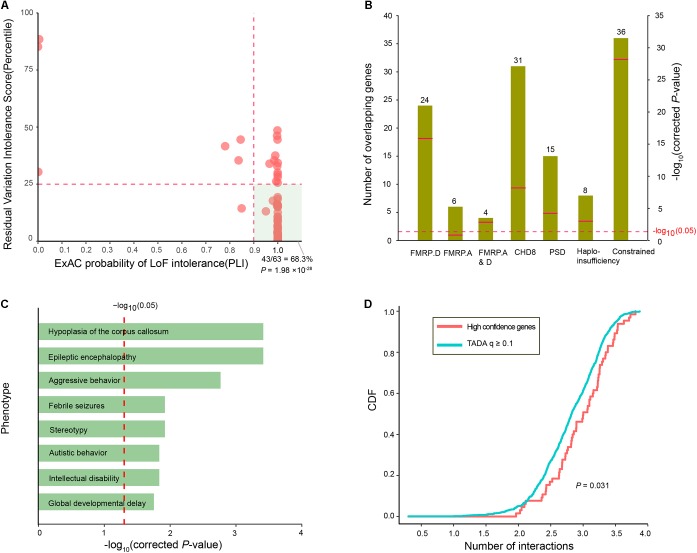
Functional characteristics of the 63 high-confidence ID risk genes. **(A)** An illustration of the intolerance of the 63 ID risk genes with pLI scores (*x*-axis) and RVIS percentiles (*y*-axis). The hot region (green area) is defined by a pLI score > 0.9 and RVIS ≤ 25th percentile. The *P*-value was calculated using the Fisher’s exact test. **(B)** Enrichment analyses of the 63 risk genes in the FMRP targets from two independent data sets, CHD8 targets, PSD genes, haploinsufficient genes and constrained gene set. Green bars represent the number of overlapping genes. Red bars indicate corrected *P*-values, which were calculated with the Fisher’s exact test. **(C)** Enrichment of 63 ID genes in human phenotypes drawn from the Human Phenotype Ontology (HPO). The x-axis represents the log_10_ of the corrected *P*-values. **(D)** The cumulative distribution function (CDF) of the number of interactions (log_10_) is depicted for 63 high-confidence risk genes relative to *q*-values ≥ 0.1. A two-sample Kolmogorov–Smirnov test was used to detect the difference.

To assess whether the ID risk genes with *q*-values < 0.1 were intolerant of functional genetic variation, we used the RVIS percentile and pLI in the ExAC to measure intolerance. There were 44 ID risk genes with RVIS values in the top 25th percentile of the most constrained genes (enrichment *P* = 7.31 × 10^-13^) and 55 risk genes with pLI values ≥ 0.9 (enrichment *P* = 2.47 × 10^-46^). In addition, 43 risk genes were preferentially enriched for “hot spot zones,” defined as genes with RVIS ≤ 25th percentile and pLI values ≥ 0.9 (enrichment *P*= 1.98 × 10^-28^, **Figure 3A**). To further characterize the function of the 63 ID risk genes with *q*-values < 0.1, we performed an enrichment test for genes encoding messenger RNAs bound by FMRP, a neuronal RNA-binding protein implicated in regulating synaptic function during normal neurogenesis. The 63 ID risk genes were strongly enriched in the FMRP-related gene sets from Darnell et al. (2011) (24 risk genes, corrected *P* = 1.58 × 10^-16^). Although the significant enrichment was not observed in the FMRP targets from Ascano et al. (2012) (6 risk genes; corrected *P* = 0.12), the enrichment in the shared set of FMRP genes from the above two independent data sets still achieved statistical significance (4 risk genes; corrected *P* = 1.62 × 10^-3^). Moreover, we also found significant enrichment for several canonical functional classes involved in a wide range of neurodevelopmental phenotypes (**Figure 3B**), such as CHD8 target genes (31 risk genes, corrected *P* = 6.96 × 10^-9^), PSD genes (15 risk genes, corrected *P* = 6.50 × 10^-5^), haploinsufficient genes (8 risk genes, corrected *P* = 1.62 × 10^-3^), and constrained genes (36 risk genes, corrected *P* = 6.62 × 10^-29^).

In addition, we further assessed the phenotypic terms of enrichment of the 63 ID risk genes based on the Human Phenotype Ontology database. We found that the 63 ID risk genes were significantly enriched for eight major neurodevelopmental phenotypes in humans (all corrected *P* < 0.05; **Figure 3C** and **Supplementary Table S5**). Hypoplasia of the corpus callosum was the most highly enriched (corrected *P* = 3.86 × 10^-4^), followed by epileptic encephalopathy, aggressive behavior, febrile seizures, stereotypy, autistic behavior, ID and global developmental delay. Constrained genes or genes with missense mutations in neuropsychiatric disorders have been proposed to have more protein interactions than non-constrained genes or controls (Shohat et al., 2017). Consistent with previous hypotheses, we found that 63 ID risk genes had a significant excess of PPIs compared with genes with *q*-values ≥ 0.1 identified in the present study (*P* = 0.031, **Figure 3D**).

### Spatiotemporal Expression Profiles of ID Risk Genes Involved in Brain Development

**FIGURE 4:**
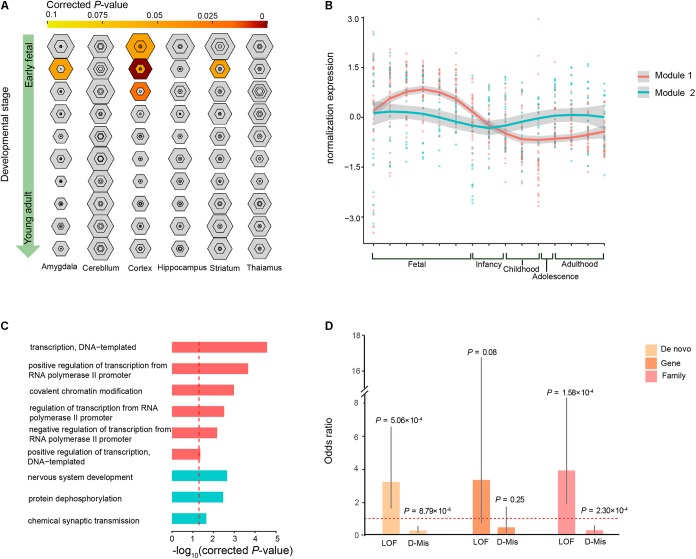
Specific expression patterns of the 63 ID risk genes in the brain. **(A)** Enrichment analysis across brain regions and development periods is depicted for different specificity index thresholds (pSIs). The outer hexagons depict pSI < 0.05, and the inner hexagons indicate a more stringent pSI. The dimension of the hexagons is scaled to the size of the gene list. Bullseyes will be color filled by corrected *P*-values calculated by Fisher’s exact test. **(B)** Illustration of the WGCNA of the 63 ID risk genes in the brain for the modules’ eigengenes (dots for different brain regions) and smooth curves for the confidence intervals (gray ranges). **(C)** GO enrichment analysis for the two modules. All *P*-values are corrected using correction for multiple comparisons. The red dotted line indicates a corrected *P* = 0.05. **(D)** Enrichment analysis of mutation class (LoF and D-Mis) from both modules at the DNM level, gene level and sample level. OR: odds ratio (module 1/module 2); *P-*values were calculated using two-sided Fisher’s exact tests. Fetal is composed of 4–8 PCW, 8–10 PCW, 10–13 PCW, 13–16 PCW, 16–19PCW, 19–24PCW, and 24–38PCW; Infancy includes 0–6 months and 6–12 months; Childhood contains 1–6 years and 6–12 years; Adolescence refers to 12–20 years; Adulthood is made up of 20–40 years, 40–60 years and over 60 years.

To investigate whether the co-expression of the 63 ID risk genes was enriched in specific tissue of human or stages of human brain development most pertinent to ID, we performed TSEA and spatiotemporal enrichment in brain using previously developed tools (Xu et al., 2014). We found that those 63 genes are enriched for brain expression and preferentially expressed in specific brain regions, in accordance with previous findings (**Supplementary Figure S3**) (Shohat et al., 2017). Across brain regions and developmental stages, we observed strong signals of association in the cortical regions during the early fetal, early mid-fetal and late mid-fetal stages (**Figure 4A**). In particular, the most significant enrichment was detected in the early mid-fetal stage (corrected *P* = 1.24 × 10^-8^). In addition, significant enrichment were also found for the amygdala and striatum during early mid-fetal stages (**Figure 4A**).

Given that our analysis pointed to the roles of the 63 ID risk genes in the context of human brain development, we wanted to further characterize the spatiotemporal expression dynamics of these genes and assess their molecular convergence on specific biological processes. We employed WGCNA to group 60 of the 63 risk genes into 2 different co-expression modules (M1 and M2) based on pairwise correlations between the gene expression profiles of the tissue samples from the HBT (**Figure 4B** and **Supplementary Table S6**). The gene expression profile of the largest module (M1), which contained 39 genes, revealed a gradual trend toward increased expression in the human brain from the embryonic to early mid-fetal periods [16–19 post-conception weeks (PCW)] and then a gradual decrease to the lowest expression at childhood. An enrichment analysis of GO terms showed that this group of genes significantly converged on covalent chromatin modification (corrected *P* = 1.04 × 10^-3^) and some transcriptional regulation, including positive regulation of transcription, DNA-templated (corrected *P* = 4.67 × 10^-2^), negative regulation of transcription from RNA polymerase II promoter (corrected *P* = 6.78 × 10^-3^) and positive regulation of transcription from RNA polymerase II promoter (corrected *P* = 2.15 × 10^-4^; **Figure 4C** and **Supplementary Table S7**). For M2, we found that 18 genes within this module were gradually decreased during the fetal and infancy periods, followed by a gradual increase in expression from the infancy to adolescence periods, reaching a stable level after adulthood. Functional annotation showed that the M2 genes were enriched for chemical synaptic transmission (corrected *P* = 0.023), protein dephosphorylation (corrected *P* = 3.46 × 10^-3^) and nervous system development (corrected *P* = 2.23 × 10^-3^; **Figure 4C** and **Supplementary Table S7**).

Gene Ontology enrichment analysis showed that some biological processes were specific to the genes of M1 or M2, implying that these two modules have a divergent etiology. We then evaluated whether the DNM number, genes with DNMs and patients harboring DNMs differed across the two types of functional DNMs (LoF and D-Mis) between M1 and M2. We found that M1 have higher prevalence of LoF mutations than did M2 (OR = 3.19, *P*= 5.06 × 10^-4^; two-tailed Fisher’s exact test), but a lower rate of D-Mis mutations was observed in M1 than in M2 (OR = 0.27, *P*= 8.79 × 10^-5^; two-tailed Fisher’s exact test; **Figure 4D**). Consistent with this observation, the burden in ID patients harboring LoF mutations was clearly higher in M1 than in M2 (OR = 3.88, *P*= 1.58 × 10^-4^; two-tailed Fisher’s exact test), while an excess of patients harboring D-Mis mutations was observed in M2 over M1 (OR = 0.28, *P*= 2.30 × 10^-4^; two-tailed Fisher’s exact test; **Figure 4D**). In addition, the frequency of genes with LoF and D-Mis mutations was not significantly different between M1 and M2, although a high proportion of LoF mutations was observed in M1 (for LoF, OR = 3.32, *P*= 0.08; for D-Mis, OR = 0.45, *P*= 0.25; two-tailed Fisher’s exact test).

## Discussion

Recent advances in genetic studies based on DNMs identified from large-scale WES/WGS analyses of ID patient cohorts allow us to further reinforce our understanding of the genetic etiology of ID (Gilissen et al., 2014; Hamdan et al., 2014; Lelieveld et al., 2016). However, the considerable genetic heterogeneity underlying ID makes it essential to prioritize causative mutations and explore new candidate genes as well as understand the relative biological processes associated with ID (Vissers et al., 2016). In this study, we employed the TADA statistical model to identify 63 high-confidence ID genes with *q*-values < 0.1, including 51 known and 12 potential ID genes, on the basis of coding DNM data sets from multiple trio-based WES/WGS studies in combination with four background DNMRs. We also observed a significant enrichment of FMRP targets and CHD8 targets among these 63 genes. Summarizing gene burden analyses in multiple metrics of evolutionary constraint suggests that the 63 risk genes are intolerant of functional genetic variations, highlighting the importance of their association with ID. Importantly, the enrichment of spatiotemporal gene expression signatures shows that ID genes were preferentially expressed in the cortex during the early fetal, early mid-fetal and late mid-fetal stages as well as amygdala and striatum during early mid-fetal stages. In particular, WGCNA analyses revealed an obvious convergence of the signals of these risk genes on similar biological processes, including synaptic function, chromatin modification and transcriptional regulation.

By excluding known ID genes, we highlighted 12 potential candidate ID genes from the 63 high-confidence ID genes. Moreover, several previous functional and association studies have pointed to the pathogenicity of most of the 12 potential candidate genes. Numerous genetics studies have identified pathogenic variants of *CACNA1A* (Epi, 2016; Luo et al., 2017), *CSNK2A1* (Trinh et al., 2017), *PPP1CB* (Gripp et al., 2016; Ma et al., 2016), *PPP2CA* (Reijnders et al., 2017), *SLC6A1* (Carvill et al., 2015; Halvorsen et al., 2016; Palmer et al., 2016; Yuan et al., 2017), and *USP7* (Zarrei et al., 2017) from large cohorts of unrelated patients who presented a wide spectrum of neurological and behavioral phenotypes of global developmental delay, attention deficit disorder, epileptic encephalopathy, macrocephaly, ID or sensory processing disorder. Several studies in model systems have provided definitive evidence of the role of partial genes in the neurodevelopmental process. Drosophila models have suggested that LoF alleles of *CACNA1A* affect synaptic transmission and neurodegeneration (Luo et al., 2017). A *SLC6A1*-knockout mouse model showed phenotypes of absence seizures or similar ADHD symptoms (Chen et al., 2015). A CRISPR/Cas9-based knockout of *USP7* in neurons clearly impaired its effect on the proper function of hypothalamic neurons (Hao et al., 2015). Expression profile analysis and immunohistochemistry revealed that *TCF7L2* is very highly expressed in the cortical, thalamic, and midbrain regions from the late gestational stage to the adult stage in mice (Nagalski et al., 2013). *TPNO2* and 71 other constrained genes formed a significantly connected subnetwork and were preferentially expressed in the hippocampal region during the early stages of brain development (Choi et al., 2016). Based on our analysis of the PPI and co-expression networks, the present study also provides compelling support for the strong functional association between the 12 potential candidate genes and the known ID genes with *q*-values < 0.3.

The finding in the present study that 15 of the 63 high-confidence ID genes were significantly associated with hypoplasia of the corpus callosum, which showed the highest enrichment (corrected *P* = 3.86 × 10^-4^), reflects the importance of the corpus callosum in ID. The corpus callosum is the largest forebrain commissure, comprising highly organized neocortical connections and functioning in bilateral movements, the development of language and handedness, and behavior and cognition (Raybaud, 2010; van der Knaap and van der Ham, 2011). The agenesis or dysgenesis of the corpus callosum has been implicated in severe ID by previous neuroradiologic studies that examined a wealth of magnetic resonance imaging (MRI) data on these patients (Schatz and Buzan, 2006; Luders et al., 2007; Aukema et al., 2009). With respect to healthy and autistic subjects, approximately 12.2% of patients with ID presented with a hypoplastic corpus callosum, as measured by the thickness and length of the corpus callosum on midsagittal T1-weighted images (Erbetta et al., 2015). An additional MRI study on a novel checklist of structural anomalies in 80 patients with unexplained mental retardation found mild to severe callosal anomalies in 28.8% of intellectually disabled patients, with a low IQ associated with the thinning of the corpus callosum (Spencer et al., 2005). In addition, a variety of abnormalities in the morphology of the corpus callosum are also found relatively frequently in children and adults with ASD, SCZ, and EE (Whitford et al., 2010; Basel-Vanagaite et al., 2013; Wolff et al., 2015).

There has been a large increase in the evidence supporting a shared genetic etiology between ID and other neuropsychiatric disorders, such as EE, ASD, DD (Vissers et al., 2016; Shohat et al., 2017). In the present study, some of the 63 risk genes were clearly implicated in EE, autistic behavior and global developmental delay (**Figure 3C**). Moreover, based on WES or WGS studies, several potential candidates from the 63 risk genes that harbored functional DNMs were frequently detected in ASD and DD (**Supplementary Table S8**). For example, the DNMs within *CACNA1A, CSNK2A1*, and *FBXO11* were recurrently detected in unrelated patients with severe DD syndromes in independent sequencing studies of larger cohorts (Deciphering Developmental Disorders, 2015, 2017). Recurrent DNMs harbored in the *SLC6A1* and *TCF7L2* genes were shared among ID, ASD, and DD (De Rubeis et al., 2014; Iossifov et al., 2014; Deciphering Developmental Disorders, 2015, 2017; Yuen et al., 2016), further highlighting the shared genetic basis of DNMs in neuropsychiatric disorders.

Recent studies using co-expression enrichment in the brain have identified the fetal development of the cortex as a point of molecular convergence for *de novo* Lof or missense mutations in ID (Harripaul et al., 2017b; Shohat et al., 2017), implying that altered cortical function is critical for ID susceptibility. Indeed, increased stability during evolution led to insufficient time for the evolution of a buffering capacity for the cerebral cortex, which is generally more intolerant of genetic perturbation (McGrath et al., 2011). The dysfunction of the cerebral cortex has been consistently implicated in neurodevelopmental disorders by multiple modalities (McGrath et al., 2011; Rubenstein, 2011; Hutsler and Casanova, 2016; Kim et al., 2016). Of the six major brain regions tested, the cortex showed the significantly enriched expression of the 63 ID risk genes identified in the present study, consistent with previous findings. Despite extensive genetic heterogeneity in ID, there is emerging evidence that ID-associated genes that are highly connected in co-expression networks or in modules converge on certain specific biological functions (Kochinke et al., 2016; Harripaul et al., 2017b; Shohat et al., 2017). A WGCNA analysis of our gene set identified two spatially and temporally specific modules associated with chromatin modification, chromatin organization and transcriptional regulation in M1 and with synaptic function in M2. The biological processes involved in ID are consistent with previous findings (Kochinke et al., 2016; Shohat et al., 2017), further emphasizing the role of convergent biological functions in ID.

## Conclusion

We provide multiple lines of evidence with function-related analyses from biological annotations, evolutionary constraints, gene co-expression and protein interaction networks that support the important role of these 63 high-confidence genes with *q*-values < 0.1 in the etiology of ID. In particular, we took advantage of a brain-specific network to define the preferential expression of ID genes in the cortex, and they point to a shared molecular basis for the synaptic function, chromatin modification and transcriptional regulation implicated in the pathogenesis of ID.

## Author Contributions

ZWL, NZ, and YZ contributed to the drafting and revision of the manuscript, data acquisition and analysis. TZ and YD contributed to data acquisition and data analysis. ZSL contributed to data acquisition and manuscript revision. XW and JW contributed to study concept and design, critical review and manuscript revision. All authors read and approved the final manuscript.

## Conflict of Interest Statement

The authors declare that the research was conducted in the absence of any commercial or financial relationships that could be construed as a potential conflict of interest.

## Footnotes

1 http://exac.broadinstitute.org/

2 https://david.ncifcrf.gov/

3 http://string-db.org/

4 http://hbatlas.org/

5 http://genetics.wustl.edu/jdlab/tsea/

6 http://genetics.wustl.edu/jdlab/csea-tool-2/

## Supplementary Material

FIGURE S1 Identification of candidate genes in ID. The number of genes with *q*-values < 0.3 in ID **(A)**, genes with *q*-values < 0.1 **(B)** and *q*-values < 0.3 **(C)** in control performed by the TADA method based on four background DNMRs are shown in the Venn diagram.

FIGURE S2 Protein–protein interaction (PPI) and co-expression network analyses of between 12 new genes and 741 known ID genes. **(A)** The histograms display the results of the permutation tests (100,000 simulations each) that assess the combined nodes and edges (connections) scores of the PPI networks. **(B)** The histograms display the results of the permutation tests (100,000 simulations each) that assess the combined nodes and edges (connections) scores of the co-expression networks. The vertical red lines indicate observed scores.

FIGURE S3 Over presentation of 63 ID risk genes across tissue types for human is demonstrated for different specificity index thresholds (pSIs).

TABLE S1 DNM information from the published literature on ID and control.

TABLE S2 Annotations for DNMs in ID and control.

TABLE S3 Information on reported known genes in ID.

TABLE S4 Prioritized genes with *q*-values < 0.3 by TADA.

TABLE S5 High-confidence ID genes are significantly enriched in Human Phenotype Ontology with an enrichment score of corrected *P* < 0.05.

TABLE S6 Conservative assessment of 63 ID risk genes and module information.

TABLE S7 Enrichment of biological processes of 63 ID risk genes in each module.

TABLE S8 Shared DNMs of ID risk genes in ASD and DD.

FILE S1 Code for permutation test performed in PPI and co-expression network.
