# Supplementary figures and images for: Prioritized High-Confidence Risk Genes for Intellectual Disability Reveal Molecular Convergence During Brain Development
Source: Front Genet. 2018 Sep 18;9:349. doi: 10.3389/fgene.2018.00349 (PMC6153320; doi:10.3389/fgene.2018.00349)

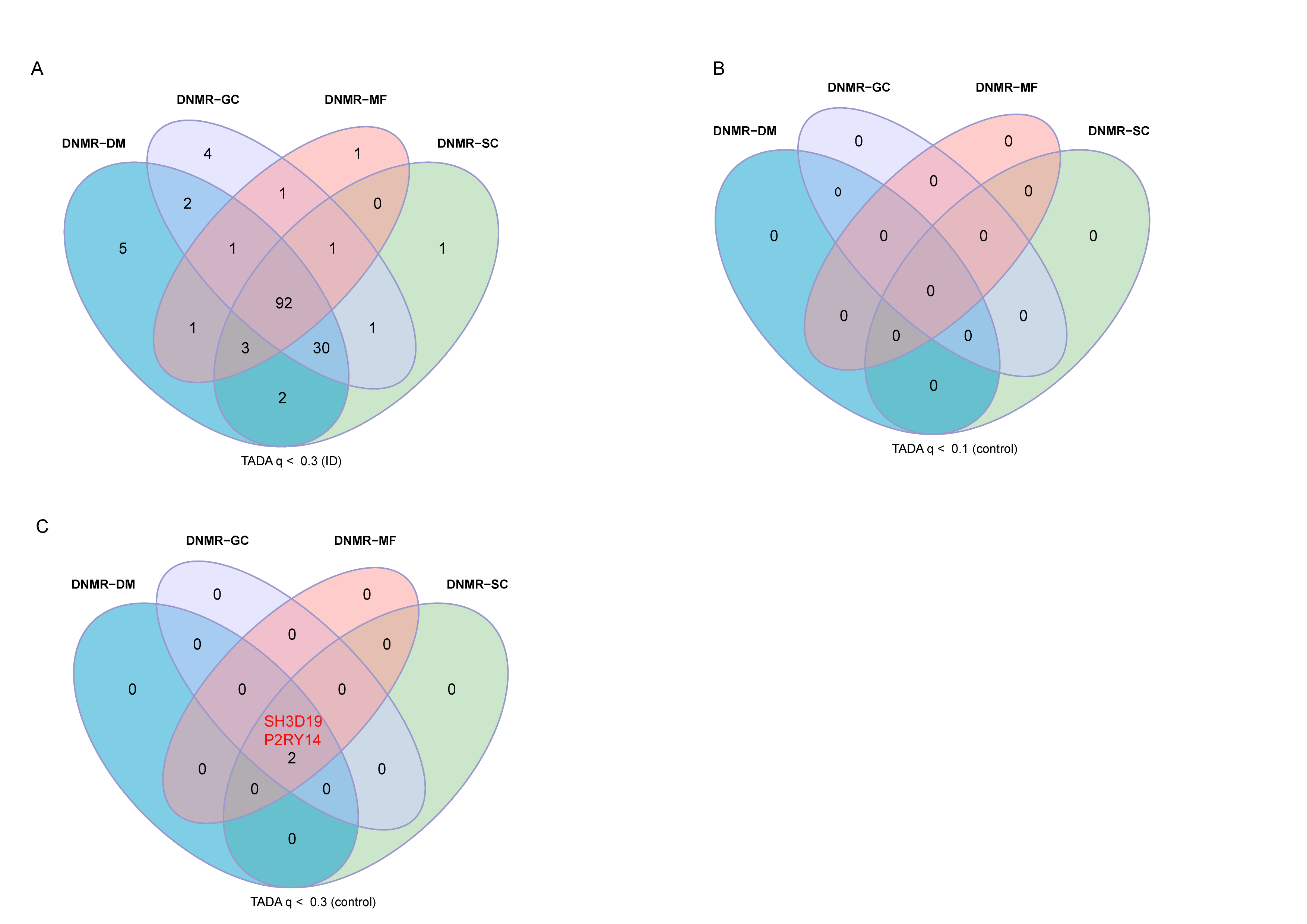

Supplement: FIGURE S1 — Identification of candidate genes in ID. The number of genes with q-values < 0.3 in ID (A), genes with q-values < 0.1 (B) and q-values < 0.3 (C) in control performed by the TADA method based on four background DNMRs are shown in the Venn diagram. [file Image_1.TIF]

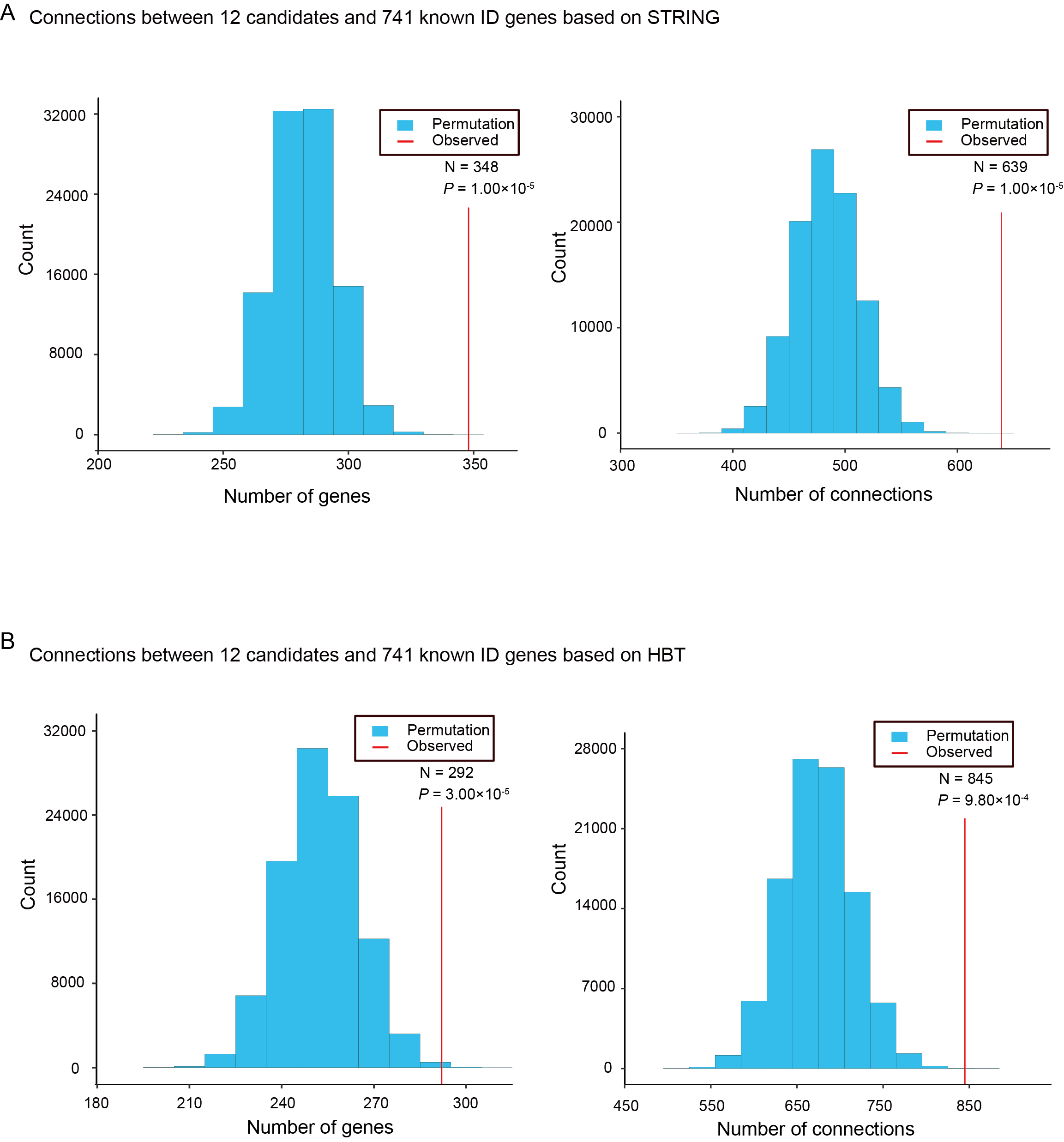

Supplement: FIGURE S2 — Protein–protein interaction (PPI) and co-expression network analyses of between 12 new genes and 741 known ID genes. (A) The histograms display the results of the permutation tests (100,000 simulations each) that assess the combined nodes and edges (connections) scores of the PPI networks. (B) The histograms display the results of the permutation tests (100,000 simulations each) that assess the combined nodes and edges (connections) scores of the co-expression networks. The vertical red lines indicate observed scores. [file Image_2.TIF]

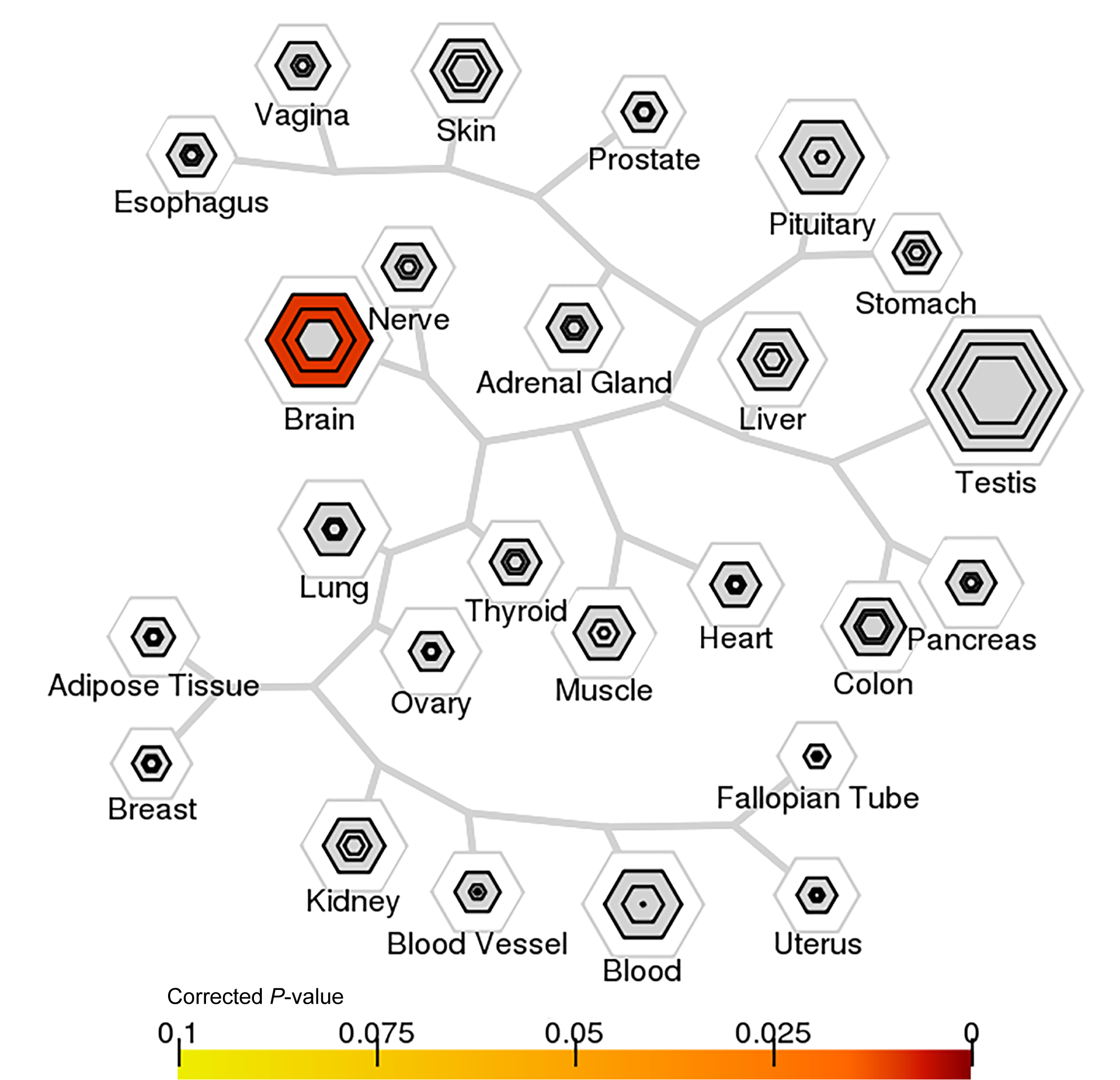

Supplement: FIGURE S3 — Over presentation of 63 ID risk genes across tissue types for human is demonstrated for different specificity index thresholds (pSIs). [file Image_3.TIF]
