# Supplementary material for: Prioritized High-Confidence Risk Genes for Intellectual Disability Reveal Molecular Convergence During Brain Development
Source: Front Genet. 2018 Sep 18;9:349. doi: 10.3389/fgene.2018.00349 (PMC6153320; doi:10.3389/fgene.2018.00349)
Supplement: TABLE S1 — DNM information from the published literature on ID and control. [file Table_1.DOCX]

**Table S1 DNM information from the published literature on ID and control**

| **Reference** | **Disease** | **Method** | **Sample information** | **Trios** | **Number of mutations** | | |
| --- | --- | --- | --- | --- | --- | --- | --- |
|  |  |  |  |  | **SNVs** | **INDELs** | **DNMs** |
| Schuurs-Hoeijmakers et al. Am J Hum Genet. 2012 [^1^](#_ENREF_1) | Intellectual disability | WES | 2 trios | 2 | 2 | 0 | 2 |
| Rauch et al. Lancet 2012 [^2^](#_ENREF_2) | Intellectual disability | WES | 51 trios | 51 | 71 | 15 | 86 |
| de Ligt et al. N Engl J Med. 2012 [^3^](#_ENREF_3) | Intellectual disability | WES | 100 trios | 50* | 49 | 9 | 58 |
| Gregor et al. Am J Hum Genet. 2013 [^4^](#_ENREF_4) | Intellectual disability | WES | 4 trios | 4 | 1 | 2 | 3 |
| Hamdan et al. Clin Genet. 2013 [^5^](#_ENREF_5) | Intellectual disability | WES | 1 trios | 1 | 1 | 0 | 1 |
| Vissers et al. Nat Genet. 2010 [^6^](#_ENREF_6) | Intellectual disability | WES | 10 trios | 10 | 8 | 1 | 9 |
| Gilissen et al. Nature. 2014 [^7^](#_ENREF_7) | Intellectual disability | WGS | 50 trios | 50 | 79 | 5 | 84 |
| Hamdan et al. PLoS Genetics 2014 [^8^](#_ENREF_8) | Intellectual disability | WES | 41trios | 41 | 67 | 14 | 81 |
| Lelieveld et al. [Nat Neurosci](https://www.ncbi.nlm.nih.gov/pubmed/?term=27479843) 2016 [^9^](#_ENREF_9) | Intellectual disability | WES | 820 trios | 818^&^ | 949 | 131 | 1,080 |
| Genome of the Netherlands Consortium. Nat Genet 2014 [^10^](#_ENREF_10) | Control | WGS | 250 families | 258 | 11020 | 291 | 11311 |
| Wendy Wong et al. Nat Commun 2016 [^11^](#_ENREF_11) | Control | WGS | 774 families (81trios are removed) | 693 | 27092 | 0 | 27092 |

* Of the 100 trios in de Ligt J et al. 2012, 50 trios are included in Gilissen et al. 2014. Overlapping samples are removed in de Ligt J et al. 2012.

^&^ Two trios in Lelieveld et al. 2016 harbored the identical DNMs to two trios in Hamdan et al. 2014 and were removed.

**References**

1. Schuurs-Hoeijmakers, J.H. *et al.* Recurrent de novo mutations in PACS1 cause defective cranial-neural-crest migration and define a recognizable intellectual-disability syndrome. *Am J Hum Genet* **91**, 1122-7 (2012).

2. Rauch, A. *et al.* Range of genetic mutations associated with severe non-syndromic sporadic intellectual disability: an exome sequencing study. *Lancet* **380**, 1674-82 (2012).

3. de Ligt, J. *et al.* Diagnostic exome sequencing in persons with severe intellectual disability. *New England Journal of Medicine* **367**, 1921-1929 (2012).

4. Gregor, A. *et al.* De novo mutations in the genome organizer CTCF cause intellectual disability. *Am J Hum Genet* **93**, 124-31 (2013).

5. Hamdan, F.F. *et al.* Parent-child exome sequencing identifies a de novo truncating mutation in TCF4 in non-syndromic intellectual disability. *Clin Genet* **83**, 198-200 (2013).

6. Vissers, L.E. *et al.* A de novo paradigm for mental retardation. *Nature genetics* **42**, 1109-1112 (2010).

7. Gilissen, C. *et al.* Genome sequencing identifies major causes of severe intellectual disability. *Nature* **511**, 344-7 (2014).

8. Hamdan, F.F. *et al.* De novo mutations in moderate or severe intellectual disability. *PLoS Genet* **10**, e1004772 (2014).

9. Lelieveld, S.H. *et al.* Meta-analysis of 2,104 trios provides support for 10 new genes for intellectual disability. *Nat Neurosci* **19**, 1194-6 (2016).

10. Genome of the Netherlands, C. Whole-genome sequence variation, population structure and demographic history of the Dutch population. *Nat Genet* **46**, 818-25 (2014).

11. Wong, W.S. *et al.* New observations on maternal age effect on germline de novo mutations. *Nat Commun* **7**, 10486 (2016).
