# Supplementary material for: Prioritized High-Confidence Risk Genes for Intellectual Disability Reveal Molecular Convergence During Brain Development
Source: Front Genet. 2018 Sep 18;9:349. doi: 10.3389/fgene.2018.00349 (PMC6153320; doi:10.3389/fgene.2018.00349)
Supplement: TABLE S3 — Information on reported known genes in ID. [file Table_3.DOCX]

**Table S3** **Information on reported known genes in ID**

| **Reference** | **Gene number** | **Gene list** | **PMID** |
| --- | --- | --- | --- |
| [Vissers](https://www.ncbi.nlm.nih.gov/pubmed/?term=Vissers%20LE%5bAuthor%5d&cauthor=true&cauthor_uid=26503795) et al. [Nat Rev Genet.](https://www.ncbi.nlm.nih.gov/pubmed/?term=26503795) 2016 | 706 | *A2ML1, ABCC9, ABCD1, ABCD4, ABHD5, ACAD9, ACO2, ACOX1, ACSF3, ACSL4, ACTB, ACTG1, ACVR1, ACY1, ADAR, ADAT3, ADCK3, ADK, ADNP, ADSL, AFF2, AGA, AGPAT2, AGTR2, AHCY, AHDC1, AHI1, AIFM1, AIMP1, AK1, AKT3, ALDH18A1, ALDH3A2, ALDH4A1, ALDH5A1, ALG1, ALG12, ALG13, ALG2, ALG3, ALG6, ALG9, ALX1, ALX4, AMPD2, AMT, ANK3, ANKH, ANKRD11, ANO10, ANTXR1, AP1S2, AP3B1, AP4B1, AP4E1, AP4M1, AP4S1, APTX, ARFGEF2, ARG1, ARHGEF6, ARHGEF9, ARID1A, ARID1B, ARL13B, ARL6, ARSE, ARX, ASL, ASNS, ASPA, ASPM, ASXL1, ASXL3, ATIC, ATP1A2, ATP2A2, ATP6AP2, ATP6V0A2, ATP7A, ATP8A2, ATR, ATRX, AUH, AUTS2, B3GALTL, B4GALT1, B4GALT7, BBS1, BBS10, BBS12, BBS2, BBS4, BBS5, BBS7, BBS9, BCKDHA, BCKDHB, BCL11A, BCOR, BCS1L, BLM, BRAF, BRWD3, BSCL2, BTD, BUB1B, C12orf57, C12orf65, C5ORF42, C7orf11, CA2, CA8, CACNG2, CAMTA1, CASK, CBL, CBS, CC2D1A, CC2D2A, CCBE1, CCDC78, CDH15, CDK5RAP2, CDKL5, CDON, CENPJ, CEP135, CEP152, CEP290, CEP41, CHAMP1, CHD2, CHD7, CHD8, CHKB, CLCNKB, CLIC2, CLN3, CLN5, CLN6, CLN8, CNTNAP2, COG1, COG6, COG7, COG8, COL4A1, COL4A2, COL4A3BP, COLEC11, COQ2, COX10, COX15, CPS1, CRADD, CRBN, CREBBP, CTCF, CTDP1, CTNNB1, CTSA, CTSD, CTTNBP2, CUBN, CUL4B, CYB5R3, D2HGDH, DARS2, DBT, DCAF17, DCX, DDHD2, DDX11, DDX3X, DEAF1, DHCR24, DHCR7, DHFR, DHTKD1, DIP2B, DKC1, DLD, DLG3, DMD, DMPK, DNAJC19, DNM1, DNMT3A, DNMT3B, DOCK8, DPAGT1, DPM1, DPP6, DPYD, DST, DYM, DYNC1H1, DYRK1A, EBP, EFTUD2, EHMT1, EIF2AK3, EIF4G1, ELOVL4, EMX2, EP300, EPB41L1, ERCC2, ERCC3, ERCC5, ERCC6, ERCC8, ERLIN2, ESCO2, ETFB, ETHE1, EXOSC3, EZH2, FAM126A, FBN1, FBXO31, FGD1, FGFR1, FGFR2, FGFR3, FH, FIGN, FKRP, FKTN, FLNA, FLVCR1, FMN2, FMR1, FOXG1, FOXP1, FOXP2, FRAS1, FTO, FTSJ1, FUCA1, GABRA1, GAD1, GALE, GALT, GAMT, GATAD2B, GATM, GCH1, GCSH, GDI1, GFAP, GJB1, GJC2, GK, GLB1, GLDC, GLI2, GLI3, GM2A, GMPPA, GMPPB, GNAO1, GNAS, GNPAT, GNS, GPC3, GPHN, GPR56, GPT2, GRIA3, GRID2, GRIK2, GRIN1, GRIN2A, GRIN2B, GRIN3B, GRM1, GSE1, GSS, GTF2H5, GUSB, HAX1, HCCS, HCFC1, HCN1, HDAC4, HDAC6, HDAC8, HECTD1, HERC1, HERC2, HESX1, HEXA, HEXB, HLCS, HOXA1, HPD, HPRT1, HRAS, HSD17B10, HSPD1, HUWE1, IDS, IDUA, IER3IP1, IFT172, IGBP1, IGF1, IKBKG, IL1RAPL1, INPP5E, IQSEC2, ISPD, ITPR1, JAG1, JAM3, KANK1, KANSL1, KAT6A, KAT6B, KCNH1, KCNJ10, KCNJ11, KCNK9, KCNQ2, KCNT1, KCTD7, KDM5C, KDM6A, KIAA0226, KIAA1033, KIAA1109, KIAA1279, KIAA2022, KIF11, KIF1A, KIF5C, KIF7, KIRREL3, KMT2A, KMT2D, KPTN, KRAS, KRBOX4, L1CAM, L2HGDH, LAMA1, LAMA2, LAMC3, LAMP2, LARGE, LARP7, LIG4, LINS, LRP2, LRPPRC, MAGEL2, MAGT1, MAN1B1, MAN2B1, MANBA, MAOA, MAP2K1, MAP2K2, MAT1A, MBD5, MBTPS2, MCCC1, MCCC2, MCOLN1, MCPH1, MECP2, MED12, MED13L, MED17, MED23, MEF2C, METTL23, MGAT2, MID1, MKKS, MLYCD, MMAA, MMACHC, MMADHC, MOCS1, MOCS2, MOGS, MPDU1, MPDZ, MRPS22, MTHFR, MTR, MTRR, MUT, MVK, MYCN, MYH9, MYO5A, MYT1L, NAA10, NAGA, NAGLU, NALCN, NBN, NDE1, NDP, NDST1, NDUFA1, NDUFA11, NDUFA12, NDUFS1, NDUFS2, NDUFS3, NDUFS4, NDUFS7, NDUFS8, NDUFV1, NEDD4L, NEU1, NF1, NFATC1, NFIA, NFIX, NHS, NIPBL, NKX2-1, NLGN3, NLGN4, NLRP3, NPHP1, NR2F1, NRAS, NRXN1, NSD1, NSDHL, NSUN2, NTRK1, OCLN, OCRL, OFD1, OPHN1, ORC1, OTC, PACS1, PAFAH1B1, PAH, PAK3, PANK2, PAX1, PAX6, PAX8, PC, PCDH19, PCGF2, PCNT, PDE4D, PDHA1, PDSS1, PDSS2, PEPD, PEX1, PEX10, PEX11B, PEX12, PEX13, PEX16, PEX19, PEX2, PEX26, PEX3, PEX5, PEX6, PEX7, PGAP1, PGAP2, PGAP3, PGK1, PHF6, PHF8, PHGDH, PHIP, PIGL, PIGN, PIGO, PIGT, PIGV, PIK3R2, PLA2G6, PLCB1, PLP1, PMM2, PNKP, PNP, POC1A, POGZ, POLG, POLR3A, POLR3B, POMGNT1, POMT1, POMT2, PORCN, POU1F1, PPOX, PPP2R1A, PPP2R5D, PPT1, PQBP1, PRODH, PRPS1, PRSS12, PSAP, PSEN1, PTCH1, PTDSS1, PTEN, PTPN11, PUF60, PURA, PUS1, PYCR1, RAB18, RAB27A, RAB39B, RAB3GAP1, RAB3GAP2, RAB40AL, RAD21, RAF1, RAI1, RARS2, RBM10, RBM28, RELN, RFT1, RIT1, RMND1, RMRP, RNASEH2A, RNASEH2B, RNASEH2C, RNASET2, ROGDI, RPGRIP1L, RPL10, RPS6KA3, RTEL1, SALL1, SATB2, SC5DL, SCN1A, SCN2A, SCN8A, SCO2, SDHA, SERAC1, SETBP1, SETD5, SGSH, SHANK2, SHANK3, SHH, SHOC2, SHROOM4, SIL1, SIN3A, SIX3, SKI, SLC12A6, SLC16A2, SLC17A5, SLC1A1, SLC1A4, SLC25A15, SLC25A22, SLC2A1, SLC33A1, SLC35A2, SLC35C1, SLC4A4, SLC6A17, SLC6A3, SLC6A8, SLC7A7, SLC9A6, SMAD4, SMARCA2, SMARCA4, SMARCB1, SMARCC2, SMARCE1, SMC1A, SMC3, SMOC1, SMPD1, SMS, SNAP29, SNIP1, SNX14, SOBP, SOS1, SOX10, SOX11, SOX2, SOX3, SOX5, SPG11, SPRED1, SPTAN1, SRCAP, SRD5A3, SRPX2, ST3GAL3, ST3GAL5, STAG1, STIL, STRA6, STT3A, STT3B, STXBP1, SUCLA2, SUOX, SURF1, SYN1, SYNE1, SYNGAP1, SYP, SYT14, TAF2, TAT, TBC1D24, TBC1D7, TBCE, TBR1, TCF4, TECR, TFAP2A, TGFBR1, TGFBR2, TGIF1, THOC6, THRB, TIMM8A, TMCO1, TMEM165, TMEM231, TMEM237, TMEM67, TMLHE, TPP1, TRAPPC11, TRAPPC9, TREX1, TRIM3, TRIO, TRMT10A, TSC1, TSC2, TSEN54, TSPAN7, TTC8, TTI2, TUBA1A, TUBA8, TUBB2B, TUBGCP6, TUSC3, TWIST1, UBE2A, UBE3A, UBE3B, UBR1, UPB1, UPF3B, USP9X, VLDLR, VPS13B, VRK1, WAC, WDR19, WDR45, WDR62, WDR81, WWOX, XPA, XPNPEP3, XYLT1, YAP1, YWHAE, YY1, ZBTB16, ZBTB18, ZDHHC15, ZDHHC9, ZEB2, ZFYVE26, ZIC2, ZMYND11, ZNF292, ZNF41, ZNF592, ZNF674, ZNF711, ZNF81* | 26503795 |
| Lelieveld et al. [Nat Neurosci](https://www.ncbi.nlm.nih.gov/pubmed/?term=27479843) 2016 | 10* | *DLG4, TCF20, PPM1D, RAC1, SMAD6, SON,* ***SOX5****, SYNCRIP, TLK2, TRIP12* | 27479843 |
| Harripaul et al. [Mol Psychiatry](https://www.ncbi.nlm.nih.gov/pubmed/?term=28397838) 2017 | 26 | *LAMC1, AFF3, AS61, PK34, TBC1D23, ZBTB11, MAP3K7, SUMF2, EXTL3, AS66, MAPK8, TET1, DMBT1, AS105, BDNF, USP44, SPATA13, SLAIN1, TRAPPC6B, VPS35, SYNRG, FBXO47, SDK2, CAPS, GPR64, MAGEA11* | 28397838 |

* Of the 10 genes in Lelieveld et all, 1 gene is included in [Vissers](https://www.ncbi.nlm.nih.gov/pubmed/?term=Vissers%20LE%5bAuthor%5d&cauthor=true&cauthor_uid=26503795) et al.
