# Supplementary material for: Prioritized High-Confidence Risk Genes for Intellectual Disability Reveal Molecular Convergence During Brain Development
Source: Front Genet. 2018 Sep 18;9:349. doi: 10.3389/fgene.2018.00349 (PMC6153320; doi:10.3389/fgene.2018.00349)
Supplement: TABLE S5 — High-confidence ID genes are significantly enriched in Human Phenotype Ontology with an enrichment score of corrected P < 0.05. [file Table_5.DOCX]

**Table S5 High-confidence ID genes are significantly enriched in Human Phenotype Ontology with an enrichment score of corrected P < 0.05**

| **Phenotype** | **Phenotype all gene Number** | **Number of Intersected genes** | ***P*-value** | **Corrected *P*-value** | **Log_10_ (*P*-value)** | **Log_10_ (corrected *P*-value)** |
| --- | --- | --- | --- | --- | --- | --- |
| Hypoplasia of the corpus callosum | 150 | 15 | 1.22E-07 | 3.86E-04 | 6.92 | 3.41 |
| Epileptic encephalopathy | 39 | 9 | 1.12E-07 | 3.86E-04 | 6.95 | 3.41 |
| Aggressive behavior | 106 | 12 | 8.11E-07 | 1.72E-03 | 6.09 | 2.77 |
| Febrile seizures | 23 | 6 | 9.46E-06 | 1.20E-02 | 5.02 | 1.92 |
| Stereotypy | 51 | 8 | 7.83E-06 | 1.20E-02 | 5.11 | 1.92 |
| Autistic behavior | 40 | 7 | 1.61E-05 | 1.46E-02 | 4.79 | 1.84 |
| Intellectual disability | 953 | 34 | 1.42E-05 | 1.46E-02 | 4.85 | 1.84 |
| Global developmental delay | 880 | 32 | 2.22E-05 | 1.76E-02 | 4.65 | 1.75 |
