# Supplementary material for: Prioritized High-Confidence Risk Genes for Intellectual Disability Reveal Molecular Convergence During Brain Development
Source: Front Genet. 2018 Sep 18;9:349. doi: 10.3389/fgene.2018.00349 (PMC6153320; doi:10.3389/fgene.2018.00349)
Supplement: TABLE S6 — Conservative assessment of 63 ID risk genes and module information. [file Table_6.DOCX]

**Table S6 Conservative assessment of 63 ID risk genes and module information**

| **Gene** | **Gene type** | **PLI** | **RVIS** | **RVIS percentile** | **mis_z** | **CHD8.Cotney** | **FMRP.Ascano** | **FMRP.Darnell** | **FMRP.Darnell & Ascano** | **Haploinsufficiency** | **Constraint**  **genes** | **psd** | **Module Colors** | **Module** |
| --- | --- | --- | --- | --- | --- | --- | --- | --- | --- | --- | --- | --- | --- | --- |
| PPP1CB | candidate:  q < 0.1 | 1 | -0.1 | 46.2 | 4.56 | Y | - | - | - | - | - | Y | blue | Module2 |
| ABCC3 | candidate:  q < 0.1 | 0 | 0.69 | 85.21 | -0.12 | - | - | - | - | - | - | - | grey | Module0 |
| CSNK2A1 | candidate:  q < 0.1 | 1 | -0.34 | 30.07 | 3.89 | - | - | - | - | - | Y | Y | turquoise | Module1 |
| TNPO2 | candidate:  q < 0.1 | 1 | -0.93 | 9.55 | 6.43 | Y | - | Y | - | - | Y | - | blue | Module2 |
| SLC6A1 | candidate:  q < 0.1 | 1 | -0.36 | 29.16 | 4.3 | - | - | Y | - | - | Y | - | blue | Module2 |
| CACNA1A | candidate:  q < 0.1 | 1 | -1.78 | 2.27 | 7.23 | Y | - | Y | - | Y | - | - | turquoise | Module1 |
| KDM2B | candidate:  q < 0.1 | 1 | -2.17 | 1.42 | 4.34 | - | - | - | - | - | Y | - | turquoise | Module1 |
| TCF7L2 | candidate:  q < 0.1 | 0.99 | -0.22 | 37.54 | 3.06 | - | - | - | - | Y | Y | - | - | - |
| USP7 | candidate:  q < 0.1 | 1 | -1.33 | 4.67 | 6.18 | Y | Y | - | - | - | Y | - | turquoise | Module1 |
| FBXO11 | candidate:  q < 0.1 | 1 | -0.69 | 15.12 | 4.03 | Y | - | - | - | - | - | - | turquoise | Module1 |
| PPP2CA | candidate:  q < 0.1 | 0.99 | -0.25 | 35.42 | 4.47 | - | - | - | - | - | - | - | blue | Module2 |
| C6orf204 | candidate:  q < 0.1 | 0 | 0.85 | 88.48 | -0.86 | - | - | - | - | - | - | - | turquoise | Module1 |
| ASXL3 | q < 0.1 | 1 | -0.67 | 15.87 | -0.94 | - | - | - | - | - | - | - | - | - |
| TUBA1A | q < 0.1 | 0.84 | -0.25 | 35.42 | 6.23 | Y | - | - | - | Y | Y | Y | blue | Module2 |
| GRIN2A | q < 0.1 | 1 | -1.46 | 3.89 | 3.8 | - | - | Y | - | - | Y | Y | blue | Module2 |
| SETBP1 | q < 0.1 | 1 | -1.12 | 6.61 | 1.9 | Y | - | - | - | - | - | - | turquoise | Module1 |
| DYRK1A | q < 0.1 | 1 | -0.42 | 25.64 | 3.37 | Y | - | - | - | - | Y | - | blue | Module2 |
| TCF20 | q < 0.1 | 1 | -2.55 | 0.85 | -0.77 | - | - | Y | - | - | - | - | turquoise | Module1 |
| KCNQ2 | q < 0.1 | 1 | -0.67 | 15.86 | 5.27 | - | - | Y | - | - | Y | Y | blue | Module2 |
| KIAA2022 | q < 0.1 | 0.95 | -0.77 | 13.1 | 0.55 | - | - | - | - | - | - | - | - | - |
| WAC | q < 0.1 | 1 | -0.87 | 10.73 | 1.57 | Y | - | - | - | - | - | - | blue | Module2 |
| COL4A3BP | q < 0.1 | 0.98 | -0.6 | 17.75 | 3.58 | Y | - | - | - | - | - | - | blue | Module2 |
| SMARCA4 | q < 0.1 | 1 | -2.85 | 0.6 | 8.36 | Y | Y | Y | Y | Y | Y | - | turquoise | Module1 |
| DLG4 | q < 0.1 | 1 | -0.67 | 15.76 | 5.4 | - | - | Y | - | Y | Y | Y | turquoise | Module1 |
| PUF60 | q < 0.1 | 0.85 | -0.71 | 14.4 | 4.51 | - | - | - | - | - | Y | - | turquoise | Module1 |
| CTNNB1 | q < 0.1 | 1 | -0.58 | 18.44 | 4.44 | Y | - | Y | - | Y | Y | Y | blue | Module2 |
| SETD5 | q < 0.1 | 1 | -0.79 | 12.6 | -0.04 | Y | - | Y | - | - | - | - | turquoise | Module1 |
| WDR45 | q < 0.1 | 0.97 | -0.27 | 33.97 | 1.69 | - | - | - | - | - | - | - | turquoise | Module1 |
| USP9X | q < 0.1 | 1 | -1.62 | 2.93 | 6.35 | Y | - | Y | - | - | Y | Y | turquoise | Module1 |
| SCN2A | q < 0.1 | 1 | -1.99 | 1.77 | 6.58 | - | - | Y | - | - | Y | - | turquoise | Module1 |
| PHIP | q < 0.1 | 1 | -1.24 | 5.49 | 5.2 | Y | - | - | - | Y | Y | - | turquoise | Module1 |
| ARID1B | q < 0.1 | 1 | -2.62 | 0.8 | 3.39 | Y | - | Y | - | - | - | - | turquoise | Module1 |
| TLK2 | q < 0.1 | 1 | -0.27 | 34.32 | 5.67 | Y | - | - | - | - | Y | - | grey | Module0 |
| POGZ | q < 0.1 | 1 | -1.53 | 3.41 | 3.36 | Y | - | - | - | - | Y | - | turquoise | Module1 |
| SOX5 | q < 0.1 | 1 | -0.96 | 9.09 | 2.82 | - | - | - | - | - | - | - | turquoise | Module1 |
| MED13L | q < 0.1 | 1 | -2.74 | 0.69 | 4.18 | Y | - | Y | - | - | Y | - | turquoise | Module1 |
| SYNCRIP | q < 0.1 | 1 | -0.56 | 19.31 | 3.94 | Y | - | - | - | Y | - | Y | grey | Module0 |
| SYNGAP1 | q < 0.1 | 1 | -1.15 | 6.23 | 7.15 | - | - | Y | - | - | Y | Y | turquoise | Module1 |
| PPP2R5D | q < 0.1 | 1 | -0.65 | 16.36 | 4.26 | - | - | - | - | - | - | Y | blue | Module2 |
| RAI1 | q < 0.1 | 1 | -3.68 | 0.25 | 1.5 | Y | - | - | - | - | - | - | turquoise | Module1 |
| EHMT1 | q < 0.1 | 1 | -1.58 | 3.13 | 2.36 | - | Y | Y | Y | - | - | - | blue | Module2 |
| SCN8A | q < 0.1 | 1 | -1.75 | 2.34 | 7.71 | - | - | Y | - | - | Y | - | blue | Module2 |
| CHD2 | q < 0.1 | 1 | -1.75 | 2.37 | 5.09 | - | - | - | - | - | Y | - | turquoise | Module1 |
| DDX3X | q < 0.1 | 1 | -0.12 | 44.54 | 5.13 | Y | - | - | - | - | Y | Y | turquoise | Module1 |
| GATAD2B | q < 0.1 | 1 | -0.07 | 48.54 | 3.4 | Y | - | - | - | - | - | - | turquoise | Module1 |
| KAT6B | q < 0.1 | 1 | -2.38 | 1.11 | 1.83 | Y | - | - | - | - | - | - | - | - |
| FOXP1 | q < 0.1 | 1 | -0.38 | 28.01 | 3 | Y | - | - | - | - | - | - | turquoise | Module1 |
| ANKRD11 | q < 0.1 | 1 | -4.38 | 0.09 | 2.76 | Y | Y | Y | Y | - | - | - | turquoise | Module1 |
| MED12 | q < 0.1 | 1 | -1 | 8.47 | 6.64 | - | Y | - | - | - | Y | - | turquoise | Module1 |
| TBR1 | q < 0.1 | 0.99 | -0.41 | 26.23 | 5.57 | - | - | - | - | - | Y | - | turquoise | Module1 |
| SON | q < 0.1 | 1 | -1.88 | 1.99 | 0.82 | Y | - | Y | - | - | - | - | turquoise | Module1 |
| SRCAP | q < 0.1 | 1 | -4.14 | 0.15 | 2.23 | Y | - | - | - | - | Y | - | turquoise | Module1 |
| MYT1L | q < 0.1 | 1 | -1.51 | 3.54 | 4.81 | - | - | Y | - | - | Y | - | turquoise | Module1 |
| STXBP1 | q < 0.1 | 1 | -0.69 | 14.97 | 5.22 | - | - | Y | - | - | Y | Y | blue | Module2 |
| AHDC1 | q < 0.1 | 1 | -1.1 | 6.98 | 4.79 | - | Y | Y | Y | - | Y | - | turquoise | Module1 |
| TRIP12 | q < 0.1 | 1 | -2.46 | 1 | 4.59 | Y | - | Y | - | - | Y | - | blue | Module2 |
| PURA | q < 0.1 | 0.85 | -0.12 | 44.54 | 5.52 | - | - | - | - | - | Y | Y | blue | Module2 |
| SATB2 | q < 0.1 | 1 | -0.87 | 10.65 | 4.75 | - | - | - | - | - | Y | - | turquoise | Module1 |
| PPM1D | q < 0.1 | 0 | -0.34 | 30.37 | 3.13 | Y | - | - | - | - | - | - | blue | Module2 |
| SLC35A2 | q < 0.1 | 0.78 | -0.16 | 41.64 | 2.33 | - | - | - | - | - | - | - | turquoise | Module1 |
| CTCF | q < 0.1 | 1 | -0.29 | 32.94 | 4.86 | Y | - | - | - | - | Y | - | turquoise | Module1 |
| FOXG1 | q < 0.1 | - | -0.25 | 35.42 | - | - | - | - | - | - | Y | - | turquoise | Module1 |
| GRIN2B | q < 0.1 | 1 | -2.41 | 1.07 | 6.74 | - | - | Y | - | - | Y | Y | turquoise | Module1 |
