# Supplementary material for: Prioritized High-Confidence Risk Genes for Intellectual Disability Reveal Molecular Convergence During Brain Development
Source: Front Genet. 2018 Sep 18;9:349. doi: 10.3389/fgene.2018.00349 (PMC6153320; doi:10.3389/fgene.2018.00349)
Supplement: TABLE S7 — Enrichment of biological processes of 63 ID risk genes in each module. [file Table_7.DOCX]

**Table S7 Enrichment of biological processes of 63 ID risk genes in each module**

| **Term** | **Count** | ***P-*value** | **Genes** | **Fold Enrichment** | **Corrected**  ***P*-value** | **Module** |
| --- | --- | --- | --- | --- | --- | --- |
| transcription, DNA-templated | 16 | 1.98E-06 | MYT1L, TCF20, SATB2, KDM2B, CSNK2A1, DDX3X, FOXG1, CHD2, GATAD2B, ARID1B, MED13L, TBR1, SRCAP, PUF60, FOXP1, SMARCA4 | 3.93 | 2.66E-05 | module1 |
| positive regulation of transcription from RNA polymerase II promoter | 11 | 1.60E-05 | PHIP, RAI1, TCF20, SATB2, DDX3X, MED12, CTCF, TCF4, TBR1, FOXP1, SMARCA4 | 5.38 | 2.15E-04 | module1 |
| covalent chromatin modification | 5 | 7.72E-05 | SATB2, CHD2, CTCF, ARID1B, SMARCA4 | 21.23 | 1.04E-03 | module1 |
| regulation of transcription from RNA polymerase II promoter | 7 | 2.28E-04 | SATB2, FOXG1, CHD2, MED13L, SRCAP, FOXP1, SMARCA4 | 7.62 | 3.07E-03 | module1 |
| negative regulation of transcription from RNA polymerase II promoter | 8 | 5.05E-04 | SATB2, KDM2B, USP9X, GATAD2B, CTCF, TCF4, FOXP1, SMARCA4 | 5.33 | 6.78E-03 | module1 |
| positive regulation of transcription, DNA-templated | 6 | 3.55E-03 | PHIP, RAI1, MED12, CTCF, TCF4, SMARCA4 | 5.59 | 4.67E-02 | module1 |
| nervous system development | 5 | 1.67E-04 | PPP2R5D, DYRK1A, SCN8A, KCNQ2, PURA | 16.25 | 2.23E-03 | module2 |
| protein dephosphorylation | 4 | 2.60E-04 | PPM1D, PPP2R5D, PPP2CA, PPP1CB | 29.62 | 3.46E-03 | module2 |
| chemical synaptic transmission | 4 | 1.69E-03 | SLC6A1, GRIN2A, KCNQ2, CTNNB1 | 15.55 | 2.23E-02 | module2 |
