# Supplementary material for: Prioritized High-Confidence Risk Genes for Intellectual Disability Reveal Molecular Convergence During Brain Development
Source: Front Genet. 2018 Sep 18;9:349. doi: 10.3389/fgene.2018.00349 (PMC6153320; doi:10.3389/fgene.2018.00349)
Supplement: TABLE S8 — Shared DNMs of ID risk genes in ASD and DD. [file Table_8.DOCX]

**Table S8 Shared DNMs of ID risk genes in ASD and DD**

| **Gene** | **Chr** | **Mutation** | **Protein change** | **Mutation type** | **Disorder** | **PMID** |
| --- | --- | --- | --- | --- | --- | --- |
| CACNA1A | chr19 | c.1441C>T | p.R481C | nonsynonymous | DD | 25533962 |
| CACNA1A | chr19 | c.2127C>A | p.F709L | nonsynonymous | DD | 28135719 |
| CACNA1A | chr19 | c.4046G>A | p.R1349Q | nonsynonymous | DD | 28135719 |
| CACNA1A | chr19 | c.2007C>G | p.D669E | nonsynonymous | DD | 28135719 |
| CACNA1A | chr19 | c.1441C>T | p.R481C | nonsynonymous | DD | 28135719 |
| CACNA1A | chr19 | c.4900G>A | p.D1634N | nonsynonymous | DD | 28135719 |
| CSNK2A1 | chr20 | c.114A>G | p.I38M | nonsynonymous | DD | 25533962 |
| CSNK2A1 | chr20 | c.526C>T | p.R176W | nonsynonymous | DD | 28135719 |
| CSNK2A1 | chr20 | c.164G>A | p.R55Q | nonsynonymous | DD | 28135719 |
| CSNK2A1 | chr20 | c.239G>A | p.R80H | nonsynonymous | DD | 28135719 |
| CSNK2A1 | chr20 | c.181T>A | p.F61I | nonsynonymous | DD | 28135719 |
| CSNK2A1 | chr20 | c.114A>G | p.I38M | nonsynonymous | DD | 28135719 |
| FBXO11 | chr2 | c.1747A>G | p.I583V | nonsynonymous | DD | 28135719 |
| FBXO11 | chr2 | c.414A>T | p.R138S | nonsynonymous | DD | 28135719 |
| FBXO11 | chr2 | c.2084-1G>A | - | splicing | DD | 28135719 |
| FBXO11 | chr2 | c.2729A>G | p.D910G | nonsynonymous | DD | 28135719 |
| FBXO11 | chr2 | c.2086_2086InsTTATAGGTC | - | frameshift | DD | 28135719 |
| SLC6A1 | chr3 | c.383C>T | p.A128V | nonsynonymous | DD | 28135719 |
| SLC6A1 | chr3 | c.187G>A | p.G63S | nonsynonymous | DD | 28135719 |
| SLC6A1 | chr3 | c.913G>A | p.A305T | nonsynonymous | DD | 28135719 |
| SLC6A1 | chr3 | c.283_283InsG | - | frameshift | DD | 28135719 |
| SLC6A1 | chr3 | c.929A>T | p.N310I | nonsynonymous | DD | 28135719 |
| SLC6A1 | chr3 | c.130C>T | p.R44W | nonsynonymous | DD | 28135719 |
| SLC6A1 | chr3 | c.331G>A | p.G111R | nonsynonymous | DD | 28135719 |
| SLC6A1 | chr3 | c.1695+2T>C | - | splicing | DD | 28135719 |
| SLC6A1 | chr3 | c.1078G>A | p.G360S | nonsynonymous | ASD | 25363760 |
| SLC6A1 | chr3 | c.863C>T | p.A288V | nonsynonymous | ASD | 25363768 |
| SLC6A1 | chr3 | c.896G>T | p.G299V | nonsynonymous | ASD | 25363768 |
| SLC6A1 | chr3 | c.1648G>A | p.G550R | nonsynonymous | ASD | 25363768 |
| SLC6A1 | chr3 | c.1015T>C | p.F339L | nonsynonymous | ASD | 27525107 |
| TCF7L2 | chr10 | c.1048C>T | p.R350X | stopgain | DD | 25533962 |
| TCF7L2 | chr10 | c.C348T | p.L116L | synonymous | DD | 28135719 |
| TCF7L2 | chr10 | c.1048C>T | p.R350X | stopgain | DD | 28135719 |
| TCF7L2 | chr10 | c.566C>T | p.S189L | nonsynonymous | DD | 28135719 |
| TCF7L2 | chr10 | c.616+1G>A | - | splicing | ASD | 25363768 |
| TCF7L2 | chr10 | c.947+1G>A | - | splicing | ASD | 25363768 |
